# Notes and Notices

**Published:** 1887-01

**Authors:** 


### NOTES AND NOTICES.

TREATMENT OF ASPHYXIA IN THE NEW-BORN.—Referring to the note with the above heading in the November number of this journal (page 414), Dr. E. A. Ballard writes us: "It recalls what an old-school M. D. told me some years ago—eight or ten. After working half-an-hour to resuscitate a new arrival considered it a hopeless case. He carried it to an adjoining room to put it out of sight of the mother, and stood a few minutes talking about a place to put it, holding it all the while by the feet, when, to his great surprise, it began to object to being longer kept head downward."

NEGATIVE AND POSITIVE EFFECTS OF HOMŒOPATHY ON GENERAL MEDICINE.—That the vast changes which have taken place in old-school treatment are mainly caused by Hahnemann's teaching, is conclusively proved by the negative and positive changes it has undergone. 1. *Negative.*—Before Hahnemann, the practice of medicine had remained almost exactly what it was for centuries previously. Theories and systems succeeded one another, but the practice of medicine remained still the same. The same weary and irrational round of bleeding, blistering, purging, vomiting, sweating, mercurial salivation was practiced through all the ages. The undeniably better results of Homœopathy forced the old school to abandon their traditional methods, and now most of them have passed into the limbo of oblivion. 2. The *positive* changes that have been effected in general medicine are, to a great extent, in the direction of Homœopathy; prescriptions have been simplified, doses have been reduced, and the very remedies and methods of Homœopathy have been largely adopted by the best and most popular writers on therapeutics. The sick and suffering have derived some benefit from this change of treatment, but they will gain infinitely greater advantage when the mass of the profession have accepted the only true rule of treatment which we owe to the genius and the labors of Hahnemann, but which their teachers, who borrow so largely from Homœopathy, carefully abstain from mentioning, or only allude to in contemptuous terms.—*Hom. League Tracts*, No. 8.

DR. B. SIMMONS, a well-known Hahnemannian, has located at 61 Catharine Street, Liverpool, England. Hahnemannians may safely recommend Dr. Simmons to their traveling patients. Dr. S. is the editor of an excellent *Cough Repertory*, now out of print.

THE MILK SUPPLY OF LARGE CITIES, AND THE IMPROPER MODE IN WHICH IT IS CONDUCTED.—Having spoken at considerable length of good cows' milk, and in what respects it resembles and differs from human milk, he referred to the abnormal conditions of milk. There is only one period, he said, at which a healthy cow will secrete unwholesome milk, and that is after parturition, when it contains colostrum. Such milk taken by the young or by invalids may produce very harmful results. It is certain that the milk will not be entirely free from colostrum corpuscles ten days after the birth of the calf, but the farmer, not knowing the pathological condition of the milk at such a period, or at least the length of time that it is affected, usually saves the sixth milking, which occurs only three days after parturition. The milk of the entire dairy may thus be contaminated by the diffusion of the colostrum through it.

In speaking of the adulteration of milk, he said that many foreign substances have been added to it, but by far the most common forms of adulteration are the removal of fat and dilution with water. Pure milk furnishes an indication of the amount of fat required by the human being in the large proportion of butter to the other solid ingredients which it contains. Besides performing certain purely mechanical offices, fat is essentially concerned in many of the chemical and vital processes of the body. As a heat producer fat stands pre-eminent, and it is mainly from the fat stored up in the body that sustenance is derived when the supply of food is temporarily cut off or the power of digestion is impaired by sickness. Furthermore, it is believed that nature has especially adapted butter fat to the digestion of infants. Altogether, therefore, it is essential in large quantities to the proper nutrition of young children.—DR. H. A. POOLER in *Medical News*.

HOMŒOPATHIC LEAGUE TRACTS.—A monthly series of tracts is published, under the above title, by Messrs. J. Beale & Sons, London. They are interesting, and, doubtless, very useful.

Information respecting the League may be obtained from the Secretary, Mr. E. H. Laurie, 16 Blandford Square, London, N. W., to whom applications for copies of the Tracts should be made.

DR. PROSPER BENDER.—We observe in the columns of *The Canadian American* a highly eulogistic notice of Dr. Bender, who, since 1883, has been settled in Boston, practicing Homœopathy. We should think Dr. Bender would be much gratified by this complimentary biography. Had we space we should republish it entire.

BEATEN AT HIS OWN GAME.—Recently a young physician of the Harvard Medical School conceived the idea of playing a joke on Miss Annie Copeland, one of the lady students of the College of Physicians and Surgeons, and at the same time rubbing out an old score he had laid up against her. His plans were carefully mapped out, and, everything being in readiness, the lady was called upon to attend a case of fracture of the leg. Somewhat astonished, she promptly answered the summons of suffering humanity, confident in her ability to sustain the dignity of the profession she had accepted. On arriving at the residence indicated, she was surprised to find her patient to be a man about forty years old, apparently suffering the most excruciating pains. Moving the covering, she discovered the fracture to be that of a wooden leg.

Nothing daunted, and without showing any evidence of her discovery, she quietly replaced the covering, said she must go for some splints and bandages, and would return immediately. She did so, bringing with her some small pieces of brass and brads, with which she at once proceeded to repair the fractured limb. The surgical operation was performed in a remarkably short space of time, and the injury left to the healing process of nature. She quietly gave the necessary directions, informed the man that he would be all right in a day or two, and that her bill was twenty-five dollars. It is scarcely necessary to say that the fee was not forthcoming, the matter being treated as a good joke. Next morning, however, Miss Copeland appeared on the scene with a constable, and, much to the chagrin of the son of Æsculapius, collected her fee. It would seem that, if the young graduates of Harvard Medical School wish to get ahead of the lady students of the College of Physicians and Surgeons, they will have to rise very early, and use more brass than Miss Copeland did in reducing the fracture of the wooden leg.—*Philadelphia Item.*

A HOPELESS CASE BEYOND A DOUBT.—Anxious Wife.—"I am afraid, doctor, you will have to send my husband to a lunatic asylum."

Omaha Doctor.—"I see no signs of mental disturbance."

"But he does such strange things."

"Does not seem possible. Tell me one of them."

"Why, this very morning I caught him way off in a corner by himself reading the President's message."—*Omaha World.*

A LIVER WITH TWO GALL-BLADDERS was recently shown before the pathological section of the Academy of Medicine of Ireland.

DR. HARLYN HITCHCOCK, of Newark, New Jersey, has moved his office from 8 Lombardy Street to 921 Broad Street. Dr. Hitchcock practices pure Homœopathy, and we wish him success.

